# Predictive value of age-specific FSH levels for IVF-ET outcome in women with normal ovarian function

**DOI:** 10.1186/s12958-015-0056-6

**Published:** 2015-06-17

**Authors:** Tingfeng Fang, Zheng Su, Liangan Wang, Ping Yuan, Ruiqi Li, Nengyong Ouyang, Lingyan Zheng, Wenjun Wang

**Affiliations:** Department of Obstetrics & Gynecology, Sun Yat-Sen Memorial Hospital, Sun Yat-Sen University, No.107 Yanjiang Xi Road, Guangzhou, 510120 P. R. China; Comprehensive Department, SunYat-Sen Memorial Hospital, Sun Yat-Sen University, No.107 Yanjiang Xi Road, Guangzhou, 510120 P. R. China

**Keywords:** Age, FSH, Premature ovarian aging (POA), IVF-ET

## Abstract

**Background:**

Most of infertile women with normal follicle stimulating hormone (FSH) levels and antral follicle count (AFC) at day 2–3 of the period, but poor IVF outcomes may occur when use of routine controlled ovarian stimulation. This paper is to evaluate the predictive value of age-specific FSH levels for IVF-ET outcomes in women with normal ovarian function.

**Methods:**

A total of 1287 women undergoing their first IVF cycles were enrolled in this retrospective study. The FSH levels and AFC of all of the women were within normal ranges (FSH ≤ 12 IU/L;AFC ≥ 5). The patients were grouped by age (younger: < 33 years, medium-aged:33–37years and older:38–41years), and within each age group, the patients were subdivided by the upper limit of the 95 % confidence interval (CI) for mean FSH levels. Patients with FSH levels equal to or greater than the upper 95 % CI of FSH in each age group were included into a premature ovarian aging (POA) subgroup (younger:FSH ≥ 7.84, medium-aged: ≥8.12 and older: FSH ≥ 8.47),the remaining patients in each age group were included into a control subgroup. The outcomes of IVF-ET were compared between the POA subgroup and the control subgroup in each age group.

**Results:**

In each age group, the total dose of gonadotropin(Gn) in the POA subgroups were significantly higher than those of the corresponding control subgroups. In the younger and medium-aged groups, women in the POA subgroups had significantly lower oocyte yields, frozen embryos, and higher rates of poor ovarian response(POR) than those in the corresponding control subgroups. When controlling for age, BMI and AFC, the multiple logistic regression analysis indicated the following: In each age group, the total dose of Gn was significantly correlated with POA; the oocyte yield was significantly related to POA only in the younger group; and in the whole age groups, the incidence of POR in the POA group was 2.719 times greater than in the control group (OR = 2.719, 95 % CI [1.598–4.625], *P* < 0.001).

**Conclusion:**

Basal FSH levels combined with age (age-specific FSH levels) can be used as a more accurate marker for the ovarian response in women with normal ovarian reserves undergoing IVF-ET, particularly in women ≤37 years old.

## Background

Because ovarian reserve is the key target of infertility treatment, its accurate evaluation is extremely important for predicting the outcomes of *in-vitro* fertilization (IVF). However, the diagnostic parameters of diminished ovarian reserve (DOR) remain controversial [1–3], it has been widely accepted that DOR can be determined when the serum basal follicle- stimulating hormone(FSH) concentration exceeds the normal range of > 10–12 IU/L [4–6].

DOR is a process that progresses with aging, revealing the variation in ovary function over time. The transition period from normal to completely degenerated ovarian reserve is called early ovarian aging, and during this period, women are asymptomatic [7, 8],However, for young women with early ovarian aging, the FSH levels and antral follicle count (AFC) at day2–3 of the period are both within normal ranges. Therefore, the use of routine controlled ovarian stimulation might lead to adverse outcomes, such as a low numbers of retrieved oocytes, high cycle cancellation rates, and low pregnancy rates. This phenomenon commonly occurs in women with unknown causes of infertility, and the incidence is approximately 9 % [9]. Even in young women with a normal basal FSH level (FSH < 10 IU/L), the number of retrieved oocytes is significantly different between women with very high and very low FSH levels [10].

However, why might poor ovarian response (POR) occur in IVF-assisted pregnant women even when FSH and AFC are both within normal ranges? This question is worth deeper investigation. A retrospective study investigating the clinical outcomes of women with normal ovarian function (FSH ≤ 12 IU/L) after controlled ovulation stimulation introduced the concept of POA. In the assessment of ovarian function using FSH, attention should be paid not only to FSH but also to the woman’s specific age. In other words, ovarian reserve can be more accurately determined by the combination of FSH and age. For relatively young women (*e.g.*, <33 years old), if FSH is at the upper limit (6.98–12 IU/L) of the normal range, indicating the age specificity of FSH, DOR might already be present. Thus, POA is defined as DOR occurring when serum FSH exceeds the upper limit of the 95 % CI of the mean FSH level in each age group. It was further confirmed that women with POA, even those with normal bFSH, might have fewer eggs or available embryos [11]. Other researchers also think that only the combination of FSH and specific age can more accurately determine ovarian reserve. In IVF-assisted pregnant women, bFSH ≤ 10 IU/L combined with specific age could better predict the number of retrieved oocytes [12]. Antral follicle count(AFC) is also an index predicting the ovarian response to controlled ovulation stimulation [13, 14]. At AFC ≤ 5, poor ovarian response to ovulation stimulation will easily occur [13, 15]. Thus, we should consider the joint effects of age-specific FSH and AFC on POA.

The objective of this study was to evaluate the predictive value of age-specific FSH levels for IVF-ET outcomes in women with normal ovarian function.

## Methods

### Study population and design

The study was conducted after receiving Ethical Committee of the Sun Yat-sen Memorial Hospital approval. The patients were provided with counseling, and signed consent forms were obtained.

We retrospectively analyzed 1,287 women who underwent their first cycle of IVF treatment in the Reproduction Center at Sun Yat-sen Memorial Hospital, Sun Yat-sen University, between January 2008 and December 2011. The POA patients were screened, and their FSH levels were analyzed. The inclusion criteria were as follows: normal ovarian function; FSH ≤ 12 IU/L and AFC ≥ 5 at days 2–5 during the period; age ≤ 41 years; and an IVF treatment regimen using a standard luteal-phase long protocol. The exclusion criteria were as follows: complicating uterine abnormalities, such as hysteromyoma and adenomyosis; polycystic ovary syndrome, thyroid disease, adrenal disease, hyperprolactinemia or other endocrine disease; and congenital genital tract malformation, pelvic tuberculosis, or ovarian tumor.

### The diagnosis of POA

The patients were divided by age into three groups: younger (<33 years old), medium-aged (33–37 years old), and older (38–41 years old) groups. The 95 % confidence interval (CI) of the FSH level was computed for each group. Based on the Barad *et al.* POA classification method, patients whose FSH levels exceeded the upper limit of the 95 % CI of the mean for each age group were included in a POA subgroup [11], According to studies of the prediction of ovary reaction using AFC [13, 15], we included patients with FSH ≤12 IU/L, AFC ≥ 5, and FSH levels equal to and greater than the upper limit of the 95 % CI of the mean into the POA subgroup in each age group, whereas those with FSH levels less than the upper limit of the 95 % CI of the mean were placed into a control subgroup.

### Ovarian stimulation, insemination, and IVF treatment

Controlled ovulation stimulation was undertaken as follows. During the mid-luteal phase of the previous cycle before starting, gonadotropin releasing hormone agonist (GnRH-a, Ipsen Pharma Biotech, France) was injected for pituitary down-regulation; 10–14 days later, at day 3–5 of the period, hormone levels were detected after pituitary down-regulation. Combined with the patient’s age and AFC, we provided recombinant follicle-stimulating hormone (rFSH) to begin follicle stimulation. The initial dose of rFSH (Gonal-F, Merck Serono, Geneva, Switzerland) was150–300 IU/day depending on patient’s AFC, during ovulation stimulation, blood E_2_, progestogen and LH were detected. The dose of gonadotropin(Gn) was adjusted depending on the patient’s ovarian response. Follicular development was monitored *via* vaginal ultrasound. If bilateral ovaries contained 3 or more follicles ≥16 mm in diameter, 2 or more follicles ≥17 mm in diameter, or 1 or more follicles ≥18 mm in diameter, and serum E_2_ was greater than the expected level consistent with the size and the number of follicles, then human chorionic gonadotropin (HCG, Livzon, Zhuhai, China or Ovidrel, Serono) was injected that night. Approximately 34–36 h later, eggs were collected. After 3–4 h, fertilization was conducted *via* routine IVF. The outcome was observed after 18 h. After further cultivation to 72 h, 2–3 high-quality embryos were transferred. The remaining available embryos were freeze-stored. The transferred embryos were supported *via* routine injection of progesterone. At 14 days after transfer, serum hCG was detected. The hCG-positive patients were preliminarily diagnosed with biochemical pregnancy. At day 30, the patients received ultrasonic examinations. Those with a gestational sac and embryo bud or cardiovascular pulsation in the womb were diagnosed with clinical pregnancy.

### Hormonal measurements

1. Blood sampling: At days 1–5 of the cycle, basic sex hormone levels, including FSH, LH, E_2_, testosterone and prolactin, were measured.
2. Processing method: After complete natural solidification, blood in vacuum sampling tubes was centrifuged at room temperature and 3000 rpm for 10 min, and an appropriate amount of serum was removed and sent for further analysis.
3. Reagents and methods: Supporting reagents and devices (Beckman coulter, USA) were used in the automatic detection, plotting of standard curves, and analysis.

### Sample size calculation

Formula for sample size estimation [16]:

$$ \mathrm{N}=\frac{\left({q}_1^{-1}+{q}_2^{-1}\right){\left({t}_{\alpha /2}+{t}_{\beta}\right)}^2{S}^2}{\delta^2} $$

According to the formula for two-group divisible design sample size estimation and using the number of oocytes retrieved as the primary outcome measurement, with an accuracy index α = 0.05 and power = 80 % in a bilateral variability test, if POA and non-POA patients in each age group were matched 1:1 in this study, the total sample size would be 344 in the younger group (POA group = 172 subjects, control group =172 subjects), 360 in the medium-aged group (POA group = 180 subjects, control group = 180 subjects), and 290 in the older group (POA group = 145 subjects, control group = 145 subjects). Thus, our sample size achieved this requirement with 650 in the younger group (POA group = 294 subjects, control group = 356 subjects) and 539 in the medium-aged group (POA group = 228 subjects, control group = 311 subjects), but the small sample size of the older group (POA group = 39 subjects, control group = 59 subjects) did not meet this requirement.

### Statistical analysis

Statistical analysis was performed using SPSS software (version 20.0, IBM Corporation, USA). Data with normal distributions and continuous variables are expressed as mean ± standard deviation; data without normal distributions are expressed as medians (interquartile ranges). Data with normal distributions were compared between groups *via* the *t*-test, while data without normal distribution were compared *via* the Mann–Whitney *U* test. Qualitative data were compared between groups by the chi-square (*χ*^2^) test. A multiple logistic regression analysis was conducted to evaluate the correlation between POA and IVF outcomes when controlling for the confounders of age, BMI, and AFC. The test level was set at *α* = 0.05. Statistical significance was set at *P* < 0.05.

## Results

### Baseline characteristics of the patients who were enrolled in the study (Tables 2, 3 and 4)

In total, 1,287 patients were included in this study. The average age was 32.08 ± 4.02 years. The average FSH was 7.85 ± 1.74 IU/L. The average BMI was 21.28 ± 2.89 kg/m2. The average AFC was 14.56 ± 5.84. The patients were divided by age into three groups: a younger group (<33 years old, 50.50 %, 650/1,287), a medium-aged group (33–37 years old, 41.88 %, 539/1,287), and an older group (38–41 years old, 7.61 %, 98/1,287).

### POA diagnosis

The diagnosis of POA, as shown in Table 1, was accomplished using FSH levels for the POA and control groups of ≥7.84 IU/L vs. < 7.84 IU/L (younger group), ≥8.12 IU/L vs. < 8.12 IU/L (medium-aged group), and ≥8.47 IU/L vs. < 8.47 IU/L (older group), respectively.

**Table 1:** FSH levels for the POA and control groups

Age(years)	FSH levels (IU/L)		
	Mean(95%CI)^a^	POA	Control
<33	7.71 (7.58–7.84)	≥7.84	<7.84
33 ~ 37	7.97 (7.82–8.12)	≥8.12	<8.12
38 ~ 41	8.14 (7.81–8.47)	≥8.47	<8.47

^a^The data are presented as mean[95 % confidence interval (CI)] for FSH levels

### The correlation between POA and IVF outcomes

Tables 2, 3 and 4 show that the incidence rates of POA were 45.23 % (294/650), 42.30 % (228/539), and 39.80 % (39/98) in the younger, medium-aged and older groups, respectively. In the POA subgroups of the younger and medium-aged groups, although FSH, FSH/LH and AFC were all normal, the number of retrieved oocytes in both POA subgroups were significantly smaller, compared to the corresponding control subgroups (12.55 ± 6.22 vs.14.86 ± 7.35, *P* < 0.05, younger group; 11.06 ± 5.79 vs. 12.81 ± 6.88, *P* < 0.05, medium-aged group). The total dose of Gn in the two POA subgroups were significantly higher than in the corresponding control subgroups (2,051.08 ± 612.17vs. 1,874.20 ± 609.07, *P* < 0.05, younger group; 2,470. 68 ± 812.84 vs. 2,121.75 ± 766.98, *P* < 0.05, medium-aged group). The incidence rates of poor ovarian response (POR) in the two POA subgroups were both significantly higher (6.46 % vs.1.42 %, younger group; 8.77%vs.4.50 %, medium-aged group). The incidence rates of pregnancy in the POA subgroups were not significantly different, compared with the corresponding control subgroups. However, the number of frozen embryos was significantly smaller in the POA subgroups (*P* < 0.05). Therefore, the difference in incidence rates of pregnancy might be manifested as the outcomes for cumulative pregnancy. The fertilization rates, the cleavage rates and the numbers of available embryos were not significantly different between the subgroups. In the medium-aged group, the E_2_ levels on D_HCG_ in the POA subgroup were significantly lower than in the control subgroup (2,804.00[1,844.50–4,765.25] vs. 3,505.00[2,276.75–4,800.00], *P* < 0.05). In the older group, the total dose of Gn was significantly higher in the POA subgroup than in the control subgroup (3,388.92 ± 1,638.73vs. 2,633.63 ± 1,000.45, *P* = 0.007); there were no significant differences in the number of retrieved oocytes, rate of POR, E_2_ level on D_HCG_ day, number of frozen embryos, fertilization rate, and pregnancy rate between the two subgroups.

**Table 2:** Baseline characteristics and IVF outcomes in the POA and control groups in women aged <33 years

Characteristics	<33 years old		*t/z(χ* ^*2*^ *)*	*P*
	Control	POA		
n	356	294		
Age(y)^a^	28.64 ± 2.49	28.92 ± 2.37	1.462	0.144
Infertility years(y)^a^	3.84 ± 2.48	3.81 ± 2.34	−0.140	0.889
BMI(kg/m^2^)^a^	21.30 ± 3.06	20.35 ± 2.27	−4.528	<0.001
FSH(IU/L)^a^	6.46 ± 1.03	9.21 ± 1.00	34.362	<0.001
LH(IU/L)^b^	3.81(2.94–4.89)	4. 78(3.62–5.82)	44.850	<0.001
E_2_(ng/L)^b^	38.00(25.90–51.00)	37.77(26.00–50.58)	0.039	0.844
FSH/LH^b^	1.76(1.32–2.18)	1.95(1.57–2.50)	20.869	<0.001
AFC(n)^a^	16.14 ± 5.82	14.58 ± 5.25	−3.554	<0.001
Total dose of Gn (IU)^a^	1,874.20 ± 609.07	2,051.08 ± 612.17	3.639	<0.001
E_2_ level on D_HCG_ (ng/L)^b^	3,818.70 (2,413.24–4,800.00)	3,563.50 (2,222.75–4,800.00)	3.107	0.078
Number of retrieved oocytes(n)^a^	14.86 ± 7.35	12.55 ± 6.22	−4.234	<0.001
Rate of POR(%)^c^	1.42(5/353)	6.46(19/294)	11.585	0.001^d^
Number of frozen embryos (n)^b^	5.00(3.00–8.00)	5.00(3.00–8.00)	3.895	0.048
Number of available embryos (n)^b^	7.00(5.00–10.00)	7.00(4.00–9.00)	3.761	0.052
Fertilization rate(%)^c^	84.62(66.67–94.44)	85.71(71.43–100.00)	1.858	0.173
Cleavage rate(%)^c^	100.00 (100.00–100.00)	100.00 (100.00–100.00)	0.221	0.638
Cancellation rate(%)^c^	0.84(3/356)	2.72(8/294)	3.415	0.065^e^
Pregnancy rate (%)^c^	55.52(196/353)	54.55(156/286)	0.061	0.805

^a^The normally distributed data are presented as the mean ± SD, including age, infertility years, BMI, FSH, AFC, total dose of Gn, and number of retrieved oocytes. The *t*-test was performed to analyze statistical significance

^b^The non-normally distributed data are presented as the median (25–75th percentile). The non-parametric Mann–Whitney test was performed to calculate the Z-score

^c^The chi-square (*χ* ^2^) test was performed to analyze statistical significance

^d^The rate of POR = (the cycles of oocyte yields ≤ 3 + the cycles cancelled egg retrieval)/the total cycles

^e^The cancellation rate only included the cycles of cancelled egg retrieval

**Table 3:** Baseline characteristics and IVF outcomes in the POA and control groups in women aged 33–37 years

Characteristics	33 ~ 37 years old		*t/z(χ* ^*2*^ *)*	*P*
	Control	POA		
n	311	228		
Age(y)^a^	34.82 ± 1.25	34.86 ± 1.32	−0.327	0.744
Infertility years(y)^a^	5.67 ± 3.65	5.43 ± 3.54	0.736	0.462
BMI(kg/m^2^)^a^	21.89 ± 2.98	21.29 ± 2.98	2.336	0.020
FSH(IU/L)^a^	6.74 ± 1.10	9.64 ± 1.05	−30.747	<0.001
LH(IU/L)^b^	3.67(2.69–4.84)	4. 47 (3.36–5.69)	26.754	<0.001
E_2_(ng/L)^b^	36.00(25.00–50.48)	37.77(24.00–51.74)	1.036	0.309
FSH/LH^b^	1.87(1.41–2.51)	2.18(1.66–2.88)	20.070	<0.001
AFC(n)^a^	15.14 ± 6.30	12.98 ± 5.29	4.313	<0.001
Total dose of Gn (IU)^a^	2,121.75 ± 766.98	2,470. 68 ± 812.84	−5.087	<0.001
E_2_ level on D_HCG_ (ng/L)^b^	3,505.00 (2,276.75–4,800.00)	2,804.00 (1,844.50–4,765.25)	7.670	0.006
Number of retrieved oocytes(n)^a^	12.81 ± 6.88	11.06 ± 5.79	3.080	0.002
Rate of POR(%)^c^	4.50(14/311)	8.77(20/228)	4.059	0.044^d^
Number of frozen embryos (n)^b^	4.00(1.00–7.00)	3.00(1.00–6.00)	3.978	0.046
Number of available embryos (n)^b^	6.00(3.00–8.00)	5.00(3.00–8.00)	2.920	0.088
Fertilization rate (%)^c^	83.33(71.43–92.31)	85.71(70.80–100.00)	2.132	0.144
Cleavage rate (%)^c^	100.00 (100.00–100.00)	100.00 (100.00–100.00)	0.177	0.674
Cancellation rate(%)^c^	0. 96(3/311)	2.60(6/228)	2.226	0.136^e^
Pregnancy rate(%)^c^	46.43(143/308)	42.79 (95/222)	0.689	0.406

^a^ The normally distributed data are presented as the mean ± SD, including age, infertility years, BMI, FSH, AFC, total dose of Gn, and number of retrieved oocytes. The *t*-test was performed to analyze statistical significance

^b^The non-normally distributed data are presented as the median (25–75th percentile). The non-parametric Mann–Whitney test was performed to calculate the Z-score

^c^The chi-square (*χ* ^2^) test was performed to analyze statistical significance

^d^The rate of POR = (the cycles of oocyte yields ≤ 3 + the cycles cancelled egg retrieval)/the total cycles

^e^The cancellation rate only included the cycles of cancelled egg retrieval

**Table 4:** Baseline characteristics and IVF outcome in POA and control groups in women with age 38–41 years old

	38 ~ 41 years old		*t/z(χ* ^*2*^ *)*	*p*
	Control	POA		
n	59	39		
Age (y)^a^	39.07 ± 1.10	38.95 ± 1.05	−0.535	0.594
Infertility years(y)^a^	6.95 ± 5.21	6.59 ± 4.08	−0.387	0.700
BMI (kg/m^2^)^a^	22.36 ± 3.28	21.62 ± 1.64	−1.481	0.142
FSH(IU/L)^a^	7.05 ± 0.97	9.79 ± 0.87	14.27	<0.001
LH(IU/L)^b^	3.39(2.48–4.23)	3.63(3.01–4.53)	−1.513	0.13
E_2_ (ng/L)^b^	35.95(29.60–49.80)	39.71(31.38–53.97)	−0.947	0.344
FSH/LH^b^	2.12(1.63–2.84)	2.84(2.13–3.20)	10.175	0.001
AFC(n)^a^	11.17 ± 5.09	9.58 ± 3.33	−1.705	0.092
Total dose of Gn (IU)^a^	2,633.63 ± 1000.45	3,388.92 ± 1638.73	2.778	0.007
E_2_ level on D_HCG_ (ng/L)^b^	2,474.20 (1,789.25–3,954.14)	2,437.00 (1,272.67–3,719.84)	0.685	0.494
Number of retrieved oocytes (n)^a^	10.26 ± 5.75	8.36 ± 4.36	−1.698	0.093
The rate of POR (%)^c^	5.08(3/59)	20.51(8/39)	5.609	0.018^d^
Number of frozen embryos (n)^b^	2.00(0.00–4.00)	2.00(0.00–4.00)	−0.470	0.638
Number of available embryos (n)^b^	4.50(3.00–7.00)	5.00(3.00–7.00)	−0.129	0.898
Fertilization rate (%)^c^	79.29(58.33–96.25)	89.44(64.39–100.00)	−1.587	0.113
Cleave rage rate (%)^c^	98.75 (100.00–100.00)	100.00 (100.00–100.0)	−1.941	0.052
Cancellation rate (%)^c^	1.70(1/59)	7.69(3/39)	2.157	0.142^e^
Pregnancy rate (%)^c^	25.86(15/58)	34.29(12/36)	0.606	0.436

^a^The normally distributed data are presented as the mean ± SD, including age, infertility years, BMI, FSH, AFC, total dose of Gn, and number of retrieved oocytes. The *t*-test was performed to analyze statistical significance

^b^The non-normally distributed data are presented as the median (25–75th percentile). The non-parametric Mann–Whitney test was performed to calculate the Z-score

^c^The chi-square (*χ* ^2^) test was performed to analyze statistical significance

^d^The rate of POR = (the cycles of oocyte yields ≤ 3 + the cycles cancelled egg retrieval)/the total cycles

^e^The cancellation rate only included the cycles of cancelled egg retrieval

The results of the multiple logistic regression analysis are shown in Table 5 and indicate that in all age groups, after controlling for the confounders of age, BMI and AFC, the total Gn dose was also significantly correlated with POA (OR = 1.328, 95 % CI [1.140–1.546], *P* < 0.001,younger group; OR = 1.535,95 % CI [1.285–1.834], *P* < 0.001,medium-aged group; OR = 1.939,95 % CI [1.212–3.102], *P* = 0.006,old group;). However, the number of retrieved oocytes was significantly correlated with POA only in the younger group (OR = 0.958, 95 % CI [0.933–0.984], *P* = 0.002).

**Table 5:** The multiple logistic regression for the relationship between POA and main IVF outcomes in each age group

	<33 years			33 ~ 37 years			38 ~ 41 years		
	B	*P*	OR (95 % CI)	B	*P*	OR (95 % CI)	B	*P*	OR (5 % CI)
Age	0.048	0.181	1.049(0.978–1.125)	−0.069	0.348	0.933(0.807–1.079)	−0.375	0.114	0.687(0.431–1.095)
BMI	−0.185	0.000	0.831(0.776–0.891)	−0.090	0.006	0.914(0.857–0.975)	−0.125	0.193	0.882(0.730–1.065)
AFC	−0.019	0.268	0.981(0.949–1.015)	−0.032	0.086	0.969(0.934–1.004)	−0.050	0.384	0.951(0.850–1.064)
Number of retrieved oocytes	−0.043	0.002	0.958(0.933–0.984)	−0.015	0.348	0.985(0.954–1.017)	−0.036	0.489	0.965(0.872–1.068)
Total dose of Gn^a^	0.283	<0.001	1.328(1.140–1.546)	0.428	<0.001	1.535(1.285–1.834)	0.662	0.006	1.939(1.212–3.102)

^a^Gn are transformed into ordinal variable according to the median (25–75th percentile)

### The correlation between POA and POR

The multiple logistic regression analysis of the correlation between POA and POR in the whole age groups after controlling for the confounders of age, BMI and AFC, as shown in Table 6, revealed that the incidence of POR in the POA subgroup was 2.719 times greater than in the control subgroup (OR = 2.719, 95 % CI [1.598–4.625], *P* < 0.001).

**Table 6:** The multiple logistic regression for low ovarian response versus POA

	B	S.E.	Wald	*P*	OR 95 % CI
Age	0.088	0.035	6.300	0.012	1.092(1.019–1.170)
BMI	−0.005	0.046	0.011	0.917	0.995(0.910–1.089)
AFC	−0.073	0.028	6.833	0.009	0.929(0.879–0.982)
POA	1.002	0.271	13.603	0.000	2.719(1.598–4.625)
Concept	−5.244	1.556	11.357		

## Discussion

Ovarian reserve can be assessed by many indicators, including serum FSH, estradiol, inhibin B, anti-Müllerian hormone (AMH), and AFC. The objective with each indicator is to predict the response to ovulation stimulation and the success rate of pregnancy. “FSH level on day 2–3 of the period” is a widely used indicator of ovarian reserve. It is generally accepted that FSH level can effectively predict the ovarian response to ovulation stimulation. As reported, FSH is more effective than age in predicting the IVF/ICSI ovarian response and cycle cancellation rate, whereas age is more effective in predicting the IVF pregnancy rate [17]. A 1045-case retrospective study showed that age and basal FSH could both effectively predict the number of retrieved oocytes, but age could more effectively predict the IVF pregnancy rate [18]. A meta-analysis indicated that FSH level was a key indicator of the outcome of IVF [19]. A prospective study compared basal AFC, AMH, ovarian volume, and FSH in predicting the ovarian response to ovulation stimulation, and the findings indicated that basal FSH and AFC were sensitive biomarkers for predicting ovarian response [20].

The incidence of early ovarian aging is 10 %; specifically, the incidence of premature ovarian failure is ~1 %, and the remaining women (~9 %) have a slight to medium degree of ovarian aging, namely POA [9]. The concept of POA was proposed and defined as an FSH level higher than the 95 % CI in the specific age group, combined with the occurrence of DOR. POA could account for the “unknown cause” of infertility in some infertile women. Currently, most studies have concluded that AFC is closely correlated with ovarian response. Based on the concept of POA and using the value of AFC for predicting ovarian reserve, we defined POA as FSH ≤ 12 IU/L, AFC ≥ 5, and FSH level higher than the 95 % CI in the specific age group. Our results were consistent with the study by Barad DH *et al.*, which showed that patients with POA, even with normal ovarian function, had significantly fewer retrieved oocytes than the non-POA patients in each age group.

Further experiments showed that the total dose of Gn in the POA subgroup was significantly larger than in the control subgroup at a specific age. Among the patients with POA, the incidence of POR was 6.46 % in the younger group, 8.77 % in the medium-age group. In the medium-age group, the E_2_ level on D_HCG_ was significantly less in the POA subgroup than in the control subgroup. The number of frozen embryos in the POA subgroup was significantly smaller than in the control subgroup of younger and medium-age groups. The fertilization rates, the cleavage rates were, the number of available embryos and the pregnancy rates were not significantly different between the subgroups.

Our results demonstrated that the numbers of retrieved oocytes, the total doses of Gn, and the POR rates all differed between the patients with and without POA. Even after controlling for the confounders of age, BMI and AFC, the total Gn dose was also significantly correlated with POA in each age group, and the oocyte yield was significantly correlated with POA in the younger group. We deduced that the reduction of early ovarian reserve, a decreased number of follicle pools, and induction of more severe Gn resistance likely resulted in to low ovarian response to follicle stimulation, consistent with previous studies [21, 22]. The fertilization rates and the cleavage rates were both not significantly different between the patients with and without POA in each age group, indicating that FSH could not predict the quality of eggs, consistent with previous studies [22, 23]. Moreover, the number of available embryos and the pregnancy rates decreased among patients with POA. Thus, we deduced that the early elevation of FSH might reduce the number of available embryos, in turn resulting in a reduced number of ET embryos, thereby reducing the clinical pregnancy rate. In the younger and medium-age groups, the number of frozen embryos was significantly less in patients with POA than in the non-POA group; thus, the difference in incidence rates of pregnancy might have been manifested as the outcomes of cumulative pregnancy.

Our study indicated again that FSH was effective in predicting the ovarian response to follicle stimulation. However, this study was also different from some previous studies [24, 25]. In our study, we included those infertile women with normal FSH and AFC. However, because all of the cases exceeded the upper limit of the age-specific 95 % CI for FSH, the number of retrieved oocytes and the ovarian response were reduced, leading to poor outcomes of IVF. Our study showed that, in the medium-age and older groups, the incidence rates of POR in patients with POA were 8.77 % and 20.51 %, respectively. Thus, if early diagnosis of POA is possible, more information about ovarian reserve could be obtained prior to IVF, and an individualized ovulation-stimulating strategy could be promptly undertaken depending on the ovarian age; with this strategy, an appropriate number of eggs could be obtained, and the cycle cancellation rate and the incidence rate of POR could be reduced, all of which would improve the clinical pregnancy rate and the clinical outcomes of IVF.

A limitation of this study was the small sample size of patients in the older group, which might have resulted from the smaller number of older women with FSH levels <12 IU/L; this limitation rendered certain significant differences difficult to assess. Another limitation was the retrospective design. Although in the younger and medium-aged groups, the sample size met the requirement for a two-group divisible design, a randomized prospective study is necessary. AMH is a relatively new marker for ovarian reserve evaluations that has been validated in many studies [26–28]. Whether the age-specific FSH level is consistent with changes in AMH and whether AMH combined with the age-specific FSH level might be a new, more accurate marker for predicting ovarian reserve merit further study.

## Conclusion

For women with normal ovarian function who plan to receive follicle-stimulating *in vitro* fertilization, particularly those ≤37 years old, basal FSH levels combined with age (age-specific FSH levels) can be used as a more accurate marker to evaluate ovarian reserve.

## Abbreviations

AFC: Antral follicle counts
AMH: Anti-Müllerian hormone
CI: Confidence interval
FSH: Follicle stimulating hormone
Gn: Gonadotropin
GnRH: Gonadotropin-releasing hormone
hCG: Human chorionic gonadotropin
OR: Odds ratio
POA: Premature ovarian aging
E2: Estradiol
POR: Poor ovarian response
